# Over, and Underexpression of Endothelin 1 and TGF-Beta Family Ligands and Receptors in Lung Tissue of Broilers with Pulmonary Hypertension

**DOI:** 10.1155/2013/190382

**Published:** 2013-10-28

**Authors:** Norma Dominguez-Avila, Gabriel Ruiz-Castañeda, Javier González-Ramírez, Nora Fernandez-Jaramillo, Jorge Escoto, Fausto Sánchez-Muñoz, Ricardo Marquez-Velasco, Rafael Bojalil, Román Espinosa-Cervantes, Fausto Sánchez

**Affiliations:** ^1^Unidad Laguna, Universidad Autónoma Agraria Antonio Narro, 27010 Torreón, COAH, Mexico; ^2^Posgrado del Centro de Estudios Justo Sierra, Surutato, 80600 Badiraguato, SIN, Mexico; ^3^Universidad Autónoma Metropolitana Xochimilco, Calzada del Hueso 1100, Villa Quietud, 04960 Coyoacan, DF, Mexico; ^4^Posgrado Biología Experimental, Universidad Autónoma Metropolitana Iztapalapa, 09340 Iztapalapa, Mexico; ^5^FMVZ, Benemérita Universidad Autónoma de Puebla, 72482 Tecamachalco, PUE, Mexico; ^6^Departamento de Inmunología, Instituto Nacional de Cardiología Dr. Ignacio Chávez, Juan Badiano No. 1, Col. Sección XVI, 140080 Tlalpan, DF, Mexico

## Abstract

Transforming growth factor beta (TGF**β**) is a family of genes that play a key role in mediating tissue remodeling in various forms of acute and chronic lung disease. In order to assess their role on pulmonary hypertension in broilers, we determined mRNA expression of genes of the TGF**β** family and endothelin 1 in lung samples from 4-week-old chickens raised either under normal or cold temperature conditions. Both in control and cold-treated groups of broilers, endothelin 1 mRNA expression levels in lungs from ascitic chickens were higher than levels from healthy birds (*P* < 0.05), whereas levels in animals with cardiac failure were intermediate. Conversely, TGF**β**2 and TGF**β**3 gene expression in lungs were higher in healthy animals than in ascitic animals in both groups (*P* < 0.05). TGF**β**1, T**β**RI, and T**β**RII mRNA gene expression among healthy, ascitic, and chickens with cardiac failure showed no differences (*P* > 0.05). BAMBI mRNA gene expression was lowest in birds with ascites only in the control group as compared with the values from healthy birds (*P* < 0.05).

## 1. Introduction

Endothelin-1 is a peptide mainly produced by endothelial cells with increased expression in pulmonary hypertension. Its action is first by a powerful vasoconstrictor effect and second as a potent mitogen that can induce proliferation in multiple cell types, including vascular smooth muscle cells, leading to remodeling of pulmonary arteries [1]. In endothelial cells, TGF*β* induces endothelin-1 expression through the activation of the ALK5/Smad3 pathway [2, 3], and both participate in pulmonary artery smooth muscle hypertrophy [4]. An increased endothelin-1 mRNA level in the lungs of broilers is a common feature of natural or induced pulmonary hypertension [5–8].

In contrast, the transforming growth factor beta (TGF*β*) family has not been documented in broilers with pulmonary hypertension. TGF*β* family are cytokines whose expression increases in various forms of acute and chronic lung disease in mammals [9]. TGF*β* family members affect various biological processes including cell proliferation, differentiation, migration, adhesion, apoptosis, and extracellular matrix (ECM) production. The TGF*β* ligands are encoded by three distinct genes (TGF*β*1, TGF*β*2, and TGF*β*3). They all elicit their cellular effects by inducing heterotetrameric complexes of type I and type II serine/threonine kinase transmembrane receptors (T*β*RI and T*β*RII). TGF*β* accessory receptors regulate access of TGF*β* family members to signaling receptors: endoglin, betaglycan, and the inhibitory pseudoreceptor BMP and activin membrane-bound inhibitor homolog (BAMBI) are the best characterized. TGF*β* accessory receptors have a short intracellular domain that lacks catalytic activity. TGF*β*2 can bind to T*β*RII only in the presence of betaglycan [10]. TGF*β* signaling is silenced by the pseudo receptor BAMBI that acts as a decoy. It is closely related to T*β*RI in the extracellular domain but has an intracellular domain that exhibits no enzymatic activity.

In normal production conditions, pulmonary hypertension in broilers accounts for 5–7% of mortality. Mortality may increase due to housing conditions, altitude, hypoxia, and cold temperature, among others. Broilers susceptible to pulmonary hypertension undergo pathophysiological progression leading to right-side chronic heart failure, pressure-induced cirrhosis of the liver, and transudation of fluid into the abdominal cavity [11–13]. Increased pulmonary artery pressure includes remodeling with hypertrophy and hyperplasia of smooth muscle cells accompanied in up to 80% of the cases by the formation of occlusive plexiform lesions (plexogenic arteriopathy) [14]. Many fast growing broilers show dilated heart and clinical signs of heart failure, but not all of these broilers develop ascites. In some instances, broilers show moderate dilation of left and right ventricles but developed massive ascites, whereas many broilers with similar or even more severe heart pathology and signs of heart failure do not develop ascites [15–17]. Whether the outcome is heart failure with or without ascites, remodeling of extracellular matrix proteins is at the core. Differences in maladaptive structural changes in the major arteries and pathological remodeling in veins are observed in ascitic birds [18].

We think that differences in progression of disease in broilers may start in lung vascular endothelium. Thus, we hypothesized expression differences of endothelin-1, TGF-*β*1, TGF-*β*2, and TGF-*β*3, of their receptors T*β*RI, T*β*RII, and of their pseudoreceptor BAMBI in lung tissue of broilers with ascites, with right cardiac failure but without ascites and in healthy broilers. To evaluate early development of ascites and signs of pulmonary hypertension, we decided to grow broilers under normal and cold temperature. Therefore, we determined mRNA expression of these genes in lung samples from 4-week-old broilers raised either under normal or cold temperature conditions.

## 2. Material and Methods

Fifty-one 1-day-old male broiler chicks of a commercial strain (Arbor Acres, Tyson Foods, Torreon, COAH, Mexico) were transported to our growth facilities (Saltillo, COAH, Mexico), divided in two groups, and housed in two rooms.

The two groups were maintained with continuous lighting feed and tap water. All chickens were supplied adlibitum with a commercial mash broiler starter diet until 21 d of age and were fed a mash broiler grower diet thereafter. The birds were monitored daily for the presence of disease, weighed every week, and postmortem examination was performed in all mortalities. The first three-week birds in both groups were raised according to recommended temperatures. From day 22 to day 28, temperature was gradually reduced from 22°C to 14°C in a group (cold group). In the meantime, birds in the other group (control group) remained within recommended temperatures (21-22°C).

On days 28–30, all birds were killed by cervical dislocation, necropsied, and classed for ascites status as follows: healthy (normal, no sign of heart or liver involvement, no fluid in abdominal cavity) and cardiac failure (from mild to severe heart enlargement with flaccidity and possible hydropericardium), and ascitic (overt right ventricular hypertrophy, copious abdominal fluid with, or without fibrin clots). This classification was performed by 3 observers during necropsies and verified by anteroposterior photograph of the heart without pericardium. Samples from the apical regions of left lungs were obtained in cryotubes (1.5 mL) and snap frozen in liquid nitrogen within 15 minutes from death.

### 2.1. Total RNA Isolation and cDNA Synthesis

100 mg of lung tissue was disrupted with a tissue homogenizer (Polytron Kinematica) in 1 mL of TriPure reagent (ROCHE, Sciences, Maryland, USA). To avoid DNA carryover, DNase treatment was performed in all samples with DNase I recombinant (ROCHE, Sciences, Maryland, USA) and a second RNA purification step with 750 **µ**L of TriPure. The RNA integrity evaluation, was done visualizing ribosomal RNA 18S and 28S integrity on agarose 1.5% gels stained with ethidium bromide, whereas concentration and purity were assessed by spectrophotometry on a NanoDrop 1000 (Thermo Fisher Scientific Lafayette, CO, USA).

cDNA synthesis was performed, using 1 **µ**g of total RNA using random hexamers and the transcriptor first strand cDNA synthesis kit (ROCHE, Sciences, Maryland, USA) according to the manufacturer instructions. Finally, to evaluate DNase treatment, RT-minus reactions were performed.

### 2.2. RT-qPCR Analysis

We performed qPCR reaction using primers for each target and reference gene designed with the ProbeFinder software version 2.4 in combination with LNA probes from the Universal Probe Library Roche (Table 1). One **µ**L of cDNA, was amplified with 400 nM of each primer (Invitrogen Carlsbad California, USA), 100 nM of UPL probe (ROCHE, Rotkreuz, Switzerland), the LighCycler TaqMan Master (ROCHE, Sciences, Maryland, USA), followed by 45 cycles of 95° 10 sec., 60° 30 sec. and 72° 1 sec., in a Roche LightCycler 2.0 (ROCHE, Rotkreuz, Switzerland). The mRNA relative quantification of target genes was conducted using ACTB and GAPDH as reference genes and the 2^−ΔCt^ method. For qPCR assays quality control, determination of linearity and reproducibility was evaluated (VC < 10%) and negative amplification for RT-minus controls.

TGF-*β*1 was formerly known as TGF-*β*4 in chicken. In spite of claims to rename TGF-*β*4 as TGF-*β*1 [19, 20], it is until January 2012 that TGF-*β*1 may be located in GenBank as Gene ID: 100873157 (*Gallus gallus*).

Primers sequences used for RT-qPCR analysis of chicken mRNAs are shown in Table 1. Data are presented as means and standard errors. Statistical analysis of data was performed by the Kruskal-Wallis test, followed by Dunn's *post-hoc* comparisons and Spearman's correlations. *P* < 0.05 was considered significant. The protocol was reviewed by the Institutional Animal Care Committee prior to being approved by the University Council on Biological and Health Sciences (Universidad Autonoma Metropolitana Xochimilco, Mexico) and complied with international guidelines for research involving animal subjects.

## 3. Results

**Figure 1:**
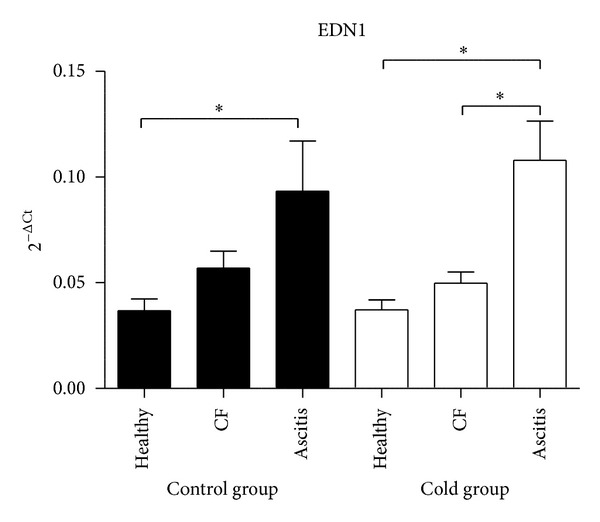
Endothelin 1 mRNA expression levels in lung tissue of ascitic broilers, broilers with cardiac failure but without ascites (CF), and healthy broilers subjected to control (solid bars) and cold-temperature treated groups (white bars). The ET-1 mRNA expression levels were normalized to those of the internal control for actin-beta (ACTB). Values are means ± SE. *N* of birds were 11, 12, and 4 for healthy, cardiac failure, and ascitic birds in control group and 9, 6, and 9 respectively, for cold group. The relative expression values were analyzed using the nonparametric Kruskal-Wallis test and Dunn's *post hoc* comparisons. *P < 0.05.

**Figure 2:**
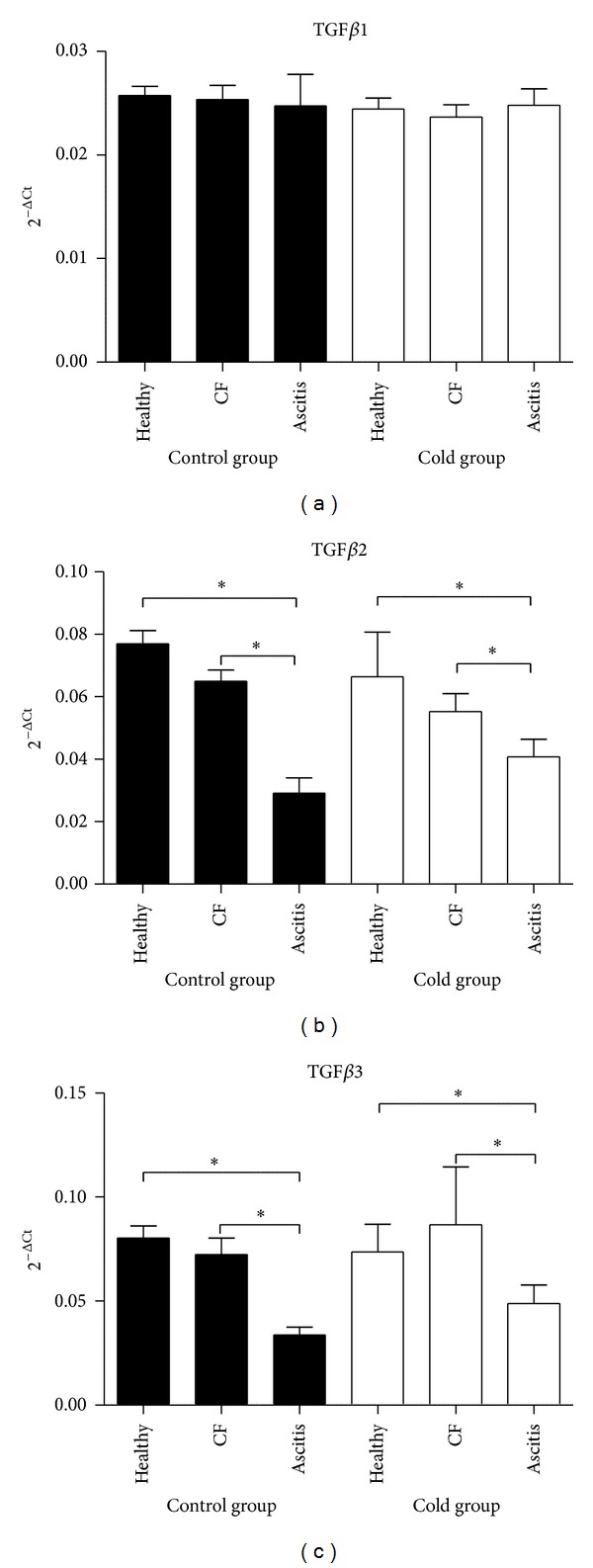
TGF*β*1, TGF*β*2, and TGF*β*3 mRNA expression levels in lung tissue of ascitic broilers, broilers with cardiac failure but without ascites (CF), and healthy broilers subjected to control (solid bars) and cold-temperature treated groups (white bars). The TGF*β*1, TGF*β*2, and TGF*β*3 mRNA expression levels were normalized to those of the internal control for beta-actin (ACTB). Values are means ± SE. *N* of birds were 11, 12, and 4 for healthy, cardiac failure, and ascitic birds in control group and 9, 6, and 9, respectively, for cold group. The relative expression values were analyzed using the nonparametric Kruskal-Wallis test and Dunn's *post hoc* comparisons. *P < 0.05.

**Figure 3:**
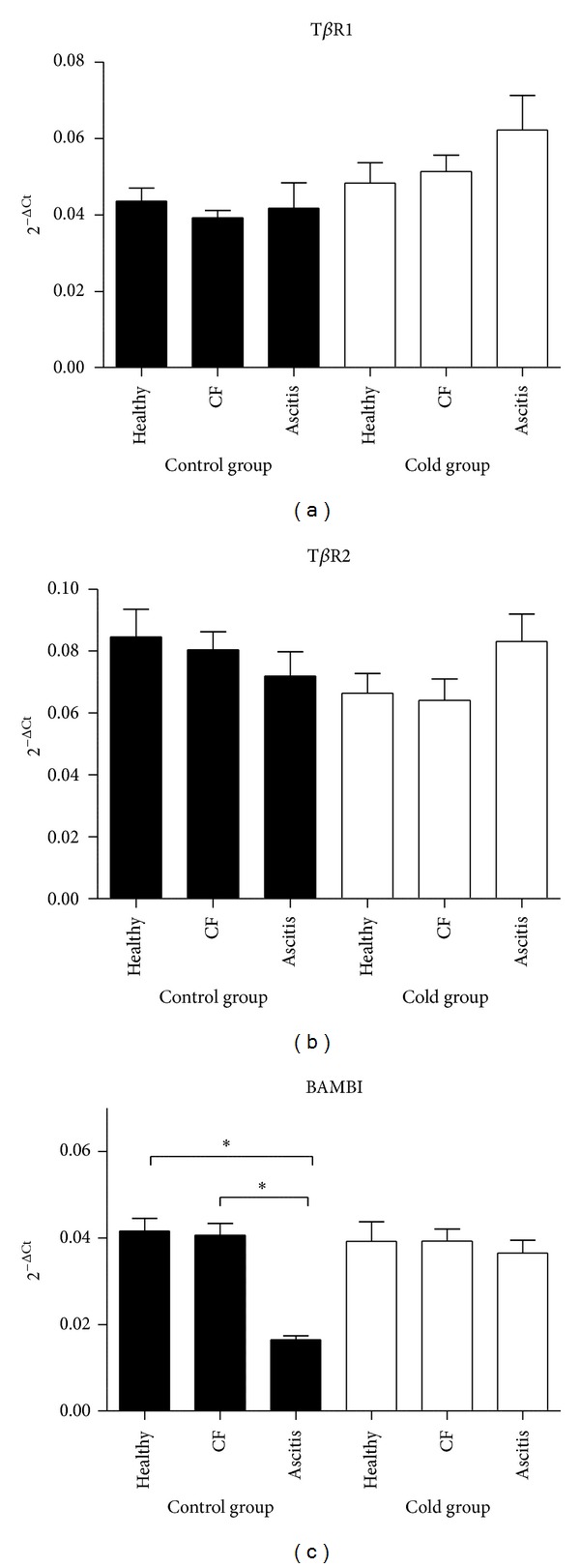
T*β*RI, T*β*RII, and BAMBI mRNA expression levels in lung tissue of ascitic broilers, broilers with cardiac failure but without ascites (CF), and healthy broilers subjected to control (solid bars) and cold-temperature treated groups (white bars). The T*β*RI, T*β*RII, and BAMBI mRNA expression levels were normalized to those of the internal control for beta-actin (ACTB). Values are means ± SE. *N* of birds were 11, 12, and 4 for healthy, cardiac failure, and ascitic birds in control group and 9, 6, and 9, respectively, for cold group. The relative expression values were analyzed using the nonparametric Kruskal-Wallis test and Dunn's *post hoc* comparisons. *P < 0.05.

Endothelin 1 mRNA expression levels in lungs from ascitic chickens were 2.5 to 2.9-fold higher than levels from healthy birds (*P* < 0.05) both in control and cold-treated groups of broilers, whereas levels in animals with cardiac failure were intermediate (Figure 1). Conversely, for TGF*β*2 and TGF*β*3 gene expression in lungs, healthy animals showed 2.1 to 2.6-fold higher expression levels than ascitic animals in both groups (*P* < 0.05), and any trend is observed for TGF*β*1 (*P* > 0.05; Figure 2). No differences were observed in T*β*RI and T*β*RII mRNA gene expressions among healthy, ascitic and chickens with cardiac failure (*P* > 0.05; Figure 3). Neither BAMBI mRNA gene expression showed differences to mention with the exception of the lowest values observed in birds with ascites as compared with healthy control group (*P* < 0.05; Figure 3). Results were also analyzed using GAPDH as another reference gene, and ACTB and GAPDH results showed good correlation (0.70 < *r* < 0.96; *P* < 0.0001) and showed the same trend.

To test associations between mRNA levels, we used Spearman's correlation test. Negative correlations for endothelin 1 with TGF*β*2 and TGF*β*3 gene expression in lungs were observed (−0.56 and −0.57; *P* < 0.05), whereas low positive correlation was found among endothelin 1 and T*β*RI (0.28; *P* < 0.05). A high correlation between TGF*β*2 and TGF*β*3 mRNA expression levels was observed (0.76; *P* < 0.05), and both variables correlated with BAMBI gene expression (0.44 and 0.45; *P* < 0.05). Finally, moderate correlations among T*β*RI and T*β*RII (0.45; *P* < 0.05) and among T*β*RII and BAMBI mRNA gene expression (0.38; *P* < 0.05) were observed. Surprisingly, not any significant correlation of TGF*β*1 mRNA gene expression with the other variables was observed (*P* > 0.05).

## 4. Discussion

We found higher endothelin 1 mRNA levels in ascitic broilers, both in control and cold treated groups, than corresponding levels for healthy broilers and broilers with cardiac failure but without ascites (*P* < 0.05). On the contrary, we found lower mRNA levels of TGF-*β*2 and TGF-*β*3 in ascitic broilers, in control, and in cold treated groups than corresponding levels in healthy broilers and with cardiac failure but without ascites (*P* < 0.05). In addition, we do not found any differences for TGF*β*1, T*β*RI, and T*β*RII mRNA gene expression among healthy, ascetic, and chickens with cardiac failure. 

As in this study, increased endothelin 1 mRNA levels have been previously reported in the lungs of broilers with natural or induced pulmonary hypertension compared to nonpulmonary hypertensive broilers [5–8]. Also, upregulated gene expression of endothelin 1 has been observed in the heart of broilers as compared with layer chickens [21] and in the heart of broiler chickens with T(3)-induced pulmonary hypertension with respect to their internal controls [22]. Endothelin 1 is produced in small amounts mainly by endothelial cells; however, in pathophysiological conditions, its production is stimulated in a large number of different cell types, including endothelial cells, vascular smooth muscle cells, fibroblasts, and inflammatory cells such as macrophages and leukocytes processes [23]. The increases of endothelin 1 mRNA levels in lung and heart are a hallmark of pulmonary hypertension syndrome in broilers.

Downregulation of mRNA levels of TGF-*β*2 and TGF-*β*3 in lungs from broilers with ascites is a novel finding both in control and cold groups. These results may be paralleled to previous findings obtained by immunohistochemistry methods. TGF*β*1 mRNA expression in alveolar macrophages from lung tissue from patients with idiopathic pulmonary fibrosis is abundant in sites of active fibrosis but not in the lung parenchyma of a patient with primary pulmonary hypertension [24]. Transforming growth factor-*β*1 decreases in remodeling hypertensive bovine pulmonary arteries, in a new-born calf's model simulating high altitude [25]. In lung tissue from patients with primary pulmonary hypertension, TGF-*β*1 immunostaining was either faint or absent in both normal and hypertensive vessels, whereas an intense, cell-associated TGF-*β*3 and a slightly less intense TGF-*β*2 immunoreactivity were observed in the media and neointimal of the hypertensive muscular arteries as compared with normal arteries of comparable size [26]. These observations suggest a distinctive role for TGF-*β*1, TGF-*β*2, and TGF-*β*3 in hypertensive pulmonary vascular remodeling.

Overall correlations among mRNA gene expression levels showed positively related TGF-*β*2 and TGF-*β*3, both negatively correlated with endothelin 1, whereas no relationship with TGF-*β*1 is observed. Other than its role as a vasoconstrictor, endothelin 1 also contributes to inflammation, as well as fibrosis during various pathophysiological processes [23]. Systemic sclerosis (SSc) has higher circulating levels of endothelin 1 and is characterized by a first inflammatory stage followed by a chronic fibrotic stage notably in the lung [27]. An avian model of SSc confirmed that as in the human disease, TGF-*β*1 has a profibrotic activity on chicken fibroblasts, but that of TGF-*β*3 and particularly TGF-*β*2 is antifibrotic and may be a candidate for SSc therapy [28, 29]. Even TGF-*β*1 in promoting fibrosis seems to have beneficial effects in SSc by inhibiting inflammation [30]. In other settings, high-density lipoproteins and TGF-*β*2 expression in endothelial cells are related to atherosclerotic plaque stability [31], and, compared to TGF-*β*1, TGF-*β*3 is associated to reduced scarring and superior healing in oral mucosal and cutaneous wounds [32, 33]. 

Concerning the inhibitory pseudoreceptor BAMBI (BMP and activin membrane-bound inhibitor homolog), we found the lowest mRNA levels in lung tissue from ascitic birds in the control groups whereas no other differences were observed. In an in vitro experiment with lung tissues obtained from COPD patients, no significant changes of TGF-*β* receptors 1 and 2 and Smad-3 expression are observed, but conversely to our results, a strong expression of BAMBI with upregulation after in vitro infection with *Haemophilus influenzae* was demonstrated [34]. Due to the small number of birds in the ascitic group (*n* = 4), BAMBI expression in controls was unexpected; thus, we analyzed lung samples from broilers (Ross) under commercial production conditions and corroborate the lower expression in the ascitic birds (data not included). It has been observed that some anti-inflammatory signals are induced during cold acclimation such as adiponectin [35] which in turn may compensate through the upregulation of BAMBI [36], thus counteracting a lowering effect of ascites on BAMBI expression from chicken in the cold group.

We speculate that an increased expression of endothelin 1 and a reduced TGF*β* signaling are related to a shortage in lung development and function. In pulmonary arterial hypertension associated to intrauterine growth restriction (IUGR), lung development and function are affected and an attenuated TGF*β* signaling is observed that may contribute to the pathological processes of IUGR-associated lung disease [37]. Also, loss of transforming growth factor beta signaling might contribute to the abnormal growth of endothelial cells in plexiform lesions in idiopathic pulmonary arterial hypertension in human [38]. Plexiform lesions are also observed in pulmonary arterial hypertension in broilers.

In conclusion, our data show an increase in endothelin-1 transcript levels as disease progresses from healthy to dilated cardiomyopathy with ascites, while a corresponding decrease in TGF-*β*2 and TGF-*β*3 is observed and seems independent of temperature conditions.

## Figures and Tables

**Table 1:** Primers used for RT-qPCR analysis of chicken mRNAs.

GENE	GenBank	PRIMERS 5′-3′	Tm °C	Amplicon bp	Probe
*EDN1 *	XM_418943.3	gccagccagagagacaagaa	60	62	29
		tgagcccagagatcttttcc	59		
*TGF-*β*1(TGF-*β*4) *	JQ423909	gtccgggctctgtacaaca	59	87	17
		ccaatactcatcgggtccat	59		
*TGF-*β*2 *	NM_001031045	ccaggttctgaaatccaaaga	59	62	9
		ccactttgctgtcaatgtaacg	60		
*TGF-*β*3 *	NM_205454	gggccctggataccaactac	60	62	25
		aagaggacgcacacagcag	60		
*T*β*R I *	NM_204246	ggaaattgctcgtcgatgtt	60	65	31
		ggtcataatatggcaactggtaatc	59		
*T*β*R II *	NM_205428	gagttcaagcaccacgacaa	59	61	7
		cctcggggatcttctcct	59		
*BAMBI *	NM_001195401	acagaggactgcacgatgttt	59	61	12
		ctgctcccttgtcctgaagt	59		
*ACTB *	NM_205518	gctctgactgaccgcgtta	60	60	76
		acgagcgcagcaatatcat	59		
*GAPDH *	NM_204305	gtcctctctggcaaagtcca	60	65	49
		accatgtagttcagatcgatgaag	59		
